# Supplementary material for: The protein expression profile of ACE2 in human tissues
Source: Mol Syst Biol. 2020 Jul 26;16(7):e9610. doi: 10.15252/msb.20209610 (PMC7383091; doi:10.15252/msb.20209610)
Supplement: Supplementary file 1 — Expanded View Figures PDF [file MSB-16-e9610-s001.pdf]

Expanded View Figures

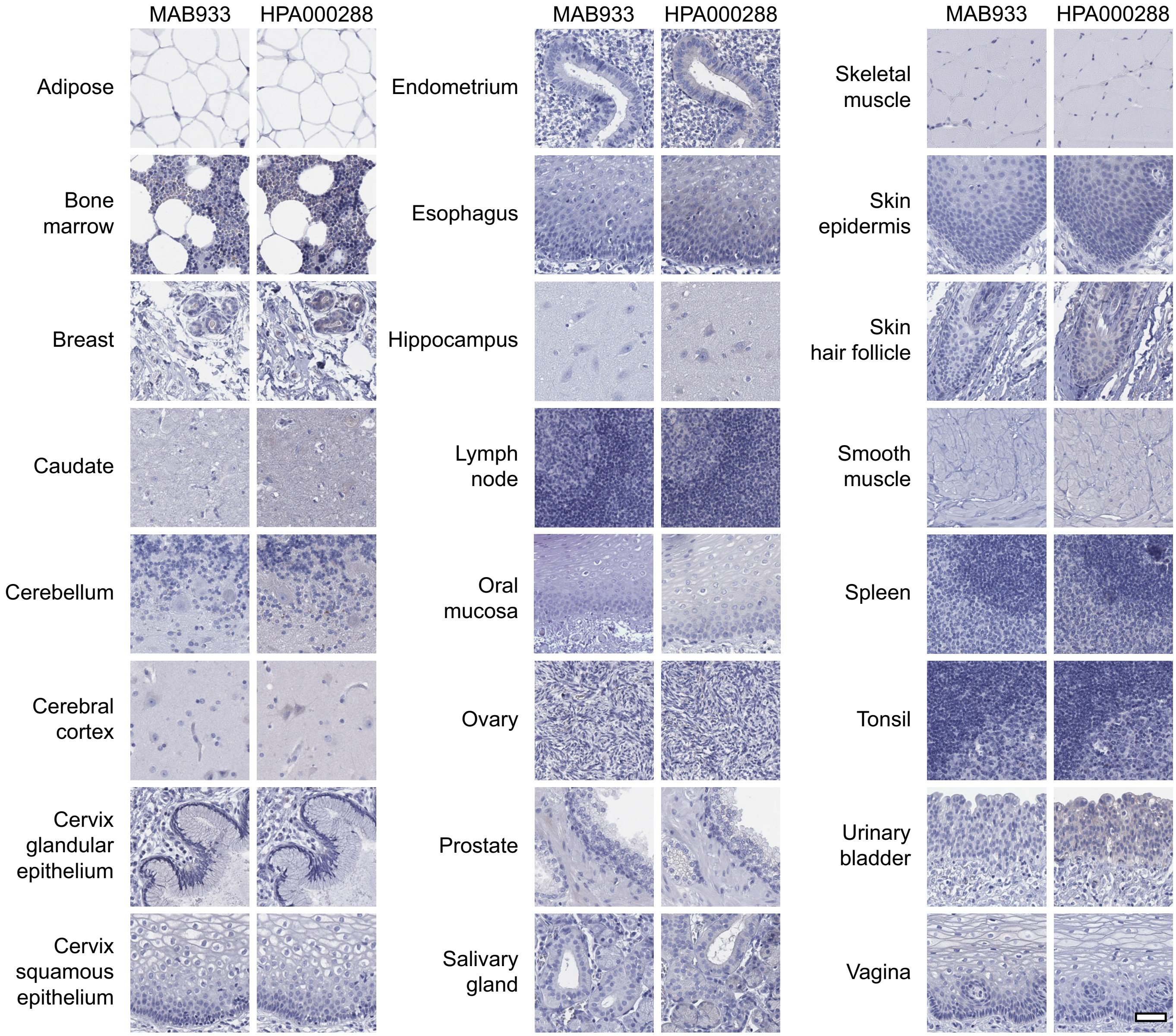

Figure EV1. Human tissues with no ACE2 protein expression based on immunohistochemistry.
Representative images of various human tissues and histological structures with no ACE2 protein expression, stained on consecutive sections with immunohistochemistry using two antibodies targeting the ACE2 protein and counterstained with hematoxylin (blue). Scale bar = 50 μm.

© 2020 The Authors
