# Supplementary material for: The protein expression profile of ACE2 in human tissues
Source: Mol Syst Biol. 2020 Jul 26;16(7):e9610. doi: 10.15252/msb.20209610 (PMC7383091; doi:10.15252/msb.20209610)
Supplement: Supplementary file 2 — Table EV1 [file MSB-16-e9610-s002.pdf]

Table Expanded View 1. Number of samples used for immunohistochemical analysis of each normal tissue type, including information on age and gender.

MAB933<br>Tissue	Total samples per tissue	Large sections	TMA samples	Female	Male	0-9 years	10-19 years	20-29 years	30-39 years	40-49 years	50-59 years	60-69 years	70-79 years	80+ years
adipose tissue	10	0	10	2	8	0	1	2	1	0	2	2	2	0
adrenal gland	5	0	5	2	3	0	0	0	0	1	3	1	0	0
appendix	8	0	8	4	4	1	1	2	0	2	1	0	1	0
bone marrow	9	0	9	3	6	0	0	0	0	1	2	4	2	0
breast	5	0	5	5	0	0	0	1	2	2	0	0	0	0
bronchioli	6	6	0	4	2	0	0	0	0	1	1	2	2	0
bronchus	13	8	5	6	7	0	0	0	0	1	2	7	3	0
caudate	8	0	8	3	5	0	1	0	1	1	2	1	2	0
cerebellum	8	0	8	5	3	0	2	1	1	0	2	1	1	0
cerebral cortex	7	0	7	1	6	0	0	0	1	0	3	1	2	0
cervix, uterine	6	0	6	6	0	0	0	1	3	1	1	0	0	0
colon	7	0	7	4	3	0	1	0	0	0	0	3	1	2
duodenum	6	0	6	3	3	0	0	0	0	0	3	2	1	0
endometrium	17	0	17	17	0	0	0	1	7	6	2	1	0	0
epididymis	10	0	10	0	10	0	0	3	2	2	2	0	1	0
esophagus	6	0	6	2	4	0	0	0	0	0	2	1	1	2
eye	1	1	0	0	1	0	0	0	0	0	0	1	0	0
fallopian tube	11	0	11	11	0	0	0	2	5	2	2	0	0	0
gallbladder	8	0	8	4	4	0	0	1	2	1	1	3	0	0
heart muscle	9	0	9	3	6	0	0	0	1	1	5	2	0	0
hippocampus	8	0	8	2	6	0	1	1	0	2	3	0	1	0
kidney	7	0	7	2	5	0	1	0	0	1	2	1	2	0
liver	8	0	8	5	3	0	0	0	1	0	4	2	1	0
lung	367	0	367	186	181	0	0	1	0	11	45	168	130	12
lymph node	11	0	11	7	4	0	0	1	2	0	7	1	0	0
nasopharynx	17	12	5	2	6	0	2	2	1	1	5	3	2	1
oral mucosa	10	0	10	6	4	0	0	0	0	0	2	4	1	3
ovary	7	0	7	7	0	0	0	1	4	2	0	0	0	0
pancreas	10	0	10	6	4	0	0	0	1	3	1	3	2	0
parathyroid gland	4	0	4	1	3	0	0	0	0	1	1	0	0	2
placenta	7	0	7	7	0	0	2	1	4	0	0	0	0	0
prostate	9	0	9	0	9	0	0	0	1	0	1	5	2	0
rectum	9	0	9	4	5	0	0	0	0	1	3	4	1	0
salivary gland	7	0	7	3	4	0	0	1	2	1	0	2	1	0
seminal vesicle	8	0	8	0	8	0	0	0	1	1	3	2	1	0
skeletal muscle	6	0	6	2	4	0	0	0	1	0	2	1	1	1
skin	15	0	15	7	8	0	1	2	1	1	3	2	5	0

small intestine	8	0	8	5	3	1	0	0	1	1	2	1	0	2
smooth muscle	9	0	9	5	4	0	2	0	2	1	1	1	0	2
spleen	7	0	7	2	5	0	2	0	0	0	2	1	2	0
stomach	15	0	15	5	10	0	0	0	0	2	6	3	4	0
testis	8	0	8	0	8	0	0	3	1	2	1	1	0	0
thyroid gland	8	0	8	5	3	0	0	2	1	1	2	2	0	0
tonsil	10	0	10	6	4	1	5	0	3	1	0	0	0	0
urinary bladder	7	0	7	2	5	0	0	0	0	1	0	3	2	1
vagina	8	0	8	8	0	0	0	0	2	5	0	1	0	0
**GRAND TOTAL**	750	27	723	370	371	3	22	29	55	61	132	243	177	28

HPA000288

Tissue	Total samples per tissue	Large sections	TMA samples	Female	Male	0-9 years	10-19 years	20-29 years	30-39 years	40-49 years	50-59 years	60-69 years	70-79 years	80+ years
adipose tissue	13	0	13	3	10	0	1	1	1	2	4	3	1	0
adrenal gland	5	0	5	2	3	0	0	0	0	1	3	1	0	0
appendix	10	0	10	6	4	0	2	3	1	2	1	0	1	0
bone marrow	11	0	11	4	7	0	0	0	0	1	2	3	4	1
breast	7	0	7	7	0	0	0	0	2	4	1	0	0	0
bronchioli	6	6	0	4	2	0	0	0	0	1	1	2	2	0
bronchus	13	8	5	6	7	0	0	0	0	1	2	7	3	0
caudate	10	0	10	4	6	0	1	0	1	2	3	1	2	0
cerebellum	8	0	8	5	3	0	2	1	0	1	2	1	1	0
cerebral cortex	10	0	10	3	7	0	1	0	1	1	4	1	2	0
cervix, uterine	8	0	8	8	0	0	0	1	2	1	2	0	1	1
colon	8	0	8	4	4	1	0	0	0	0	2	0	2	2
duodenum	8	0	8	5	3	0	0	0	0	1	2	2	3	0
endometrium	22	0	22	22	0	0	0	1	10	6	4	0	0	1
epididymis	7	0	7	0	7	0	0	2	2	1	2	0	0	0
esophagus	9	0	9	3	6	0	0	0	0	0	2	3	2	2
eye	1	1	0	0	1	0	0	0	0	0	0	1	0	0
fallopian tube	12	0	12	12	0	0	0	2	5	3	2	0	0	0
gallbladder	9	0	9	5	4	0	0	1	0	2	1	3	2	0
heart muscle	9	0	9	4	5	0	1	0	1	0	5	2	0	0
hippocampus	9	0	9	2	7	0	1	1	0	3	3	0	1	0
kidney	9	0	9	3	6	0	1	0	1	1	3	2	1	0
liver	10	0	10	6	4	0	0	0	3	0	4	1	2	0
lung	370	0	370	188	182	0	0	1	0	11	45	169	132	12
lymph node	12	0	12	7	5	0	0	0	3	0	7	2	0	0
nasopharynx	17	12	5	3	14	1	1	1	1	0	5	4	3	1
oral mucosa	8	0	8	5	3	0	0	0	0	0	2	3	1	2
ovary	8	0	8	8	0	0	0	1	3	2	0	1	1	0

pancreas	11	0	11	6	5	0	0	0	1	2	2	2	4	0
parathyroid gland	4	0	4	2	2	0	0	0	0	0	1	0	1	2
placenta	9	0	9	9	0	0	2	2	4	1	0	0	0	0
prostate	11	0	11	0	11	0	0	0	1	0	1	7	2	0
rectum	9	0	9	4	5	0	0	0	0	1	2	4	2	0
salivary gland	10	0	10	4	6	0	0	1	2	1	2	3	1	0
seminal vesicle	9	0	9	0	9	0	0	0	1	1	1	5	1	0
skeletal muscle	7	0	7	2	5	0	0	0	1	0	2	1	2	1
skin	15	0	15	6	9	0	0	0	1	2	3	2	4	3
small intestine	8	0	8	5	3	1	0	0	2	1	2	1	0	1
smooth muscle	9	0	9	5	4	0	1	0	2	2	1	2	0	1
spleen	8	0	8	2	6	0	3	0	0	0	2	1	2	0
stomach	16	0	16	6	10	0	0	0	0	2	5	3	5	1
testis	10	0	10	0	10	0	0	4	2	2	1	1	0	0
thyroid gland	9	0	9	4	5	0	0	1	2	0	3	2	1	0
tonsil	9	0	9	6	3	2	4	0	2	1	0	0	0	0
urinary bladder	6	0	6	2	4	0	0	0	0	0	0	2	3	1
vagina	9	0	9	9	0	0	0	0	1	6	0	2	0	0
**GRAND TOTAL**	798	27	771	401	397	5	21	24	59	69	142	250	195	32
