# Supplementary material for: The protein expression profile of ACE2 in human tissues
Source: Mol Syst Biol. 2020 Jul 26;16(7):e9610. doi: 10.15252/msb.20209610 (PMC7383091; doi:10.15252/msb.20209610)
Supplement: Supplementary file 4 — Table EV3 [file MSB-16-e9610-s004.pdf]

Table Expanded View 3. List of samples included in the cohort of normal lung tissues (n=360), with information on age, gender, smoking status ,and performance status, and immunohistochemical staining of ACE2.

* = 0, Fully active, able to carry on all pre-disease performance without restriction; 1, Restricted in physically strenuous activity but ambulatory and able to carry out work of a light or sedentary nature, e.g., light house work, office work; 2, Ambulatory and capable of all selfcare but unable to carry out any work activities. Up and about more than 50% of waking hours; 3, Capable of only limited selfcare, confined to bed or chair more than 50% of waking hours; 4, Completely disabled. Cannot carry on any selfcare. Totally confined to bed or chair.

Individual	Age (years)	Gender (F=female, M=male)	Smoking (C=current, F=former, N=never)	Performance status (ECOG/WHO)*	Positive (+) or negative (-) IHC staining MAB933	Positive (+) or negative (-) IHC staining HPA000288
1	78	M	N	1	-	-
2	74	M	F	1	-	-
3	74	F	C	0	-	-
4	73	M	C	1	-	-
5	74	F	F	0	-	-
6	64	M	N	0	-	-
7	64	M	C	0	-	-
8	63	F	F	0	-	-
9	61	F	C	0	-	-
10	58	F	N	0	-	-
11	83	F	F	1	-	-
12	72	F	F	0	-	-
13	71	M	C	0	-	-
14	70	F	N	0	-	-
15	70	M	F	0	-	-
16	65	M	C	0	-	-
17	61	F	F	0	-	-
18	57	F	C	1	-	-
19	47	M	C	1	-	-
20	70	M	F	1	-	-
21	80	M	F	1	-	-
22	80	M	F	0	-	-
23	78	M	C	1	-	-
24	75	F	C	1	-	-
25	74	F	F	1	-	-
26	74	F	C	2	-	-
27	71	F	F	0	-	-
28	67	M	F	0	-	-
29	65	M	C	1	-	-
30	65	M	C	0	-	-
31	64	M	C	1	-	-
32	63	M	C	2	-	-
33	62	M	F	0	-	-
34	58	F	N	0	-	-
35	52	F	C	0	-	-
36	48	M	C	1	-	-
37	46	F	C	0	-	-
38	76	F	C	0	-	-
39	75	M	N	0	-	-
40	70	M	C	0	-	-
41	67	M	F	0	-	-
42	67	F	F	0	-	-
43	66	M	F	0	-	-
44	65	F	C	0	-	-
45	53	M	C	0	-	-
46	78	F	C	0	-	-
47	76	M	N	0	-	-
48	71	M	C	1	-	-

49	66	F	C	0	-	-
50	64	F	C	0	-	-
51	64	M	C	0	-	-
52	74	M	N	0	-	-
53	74	F	C	1	-	-
54	71	F	C	1	-	-
55	71	F	C	0	-	-
56	70	F	C	0	-	-
57	70	F	C	1	-	-
58	65	M	C	1	-	-
59	64	F	F	0	-	-
60	63	M	N	1	-	-
61	62	M	F	0	-	-
62	61	F	C	1	-	-
63	58	M	C	0	-	-
64	58	M	C	1	-	-
65	57	M	C	0	-	-
66	56	F	C	0	-	-
67	56	M	C	1	-	-
68	77	M	C	1	-	-
69	77	M	F	1	-	-
70	65	F	F	1	-	-
71	67	M	C	2	-	-
72	56	F	N	0	-	-
73	79	F	F	1	-	-
74	76	F	C	0	-	-
75	74	M	F	1	-	-
76	69	F	F	1	-	-
77	66	M	F	1	-	-
78	65	M	F	0	-	-
79	64	M	F	0	-	-
80	56	M	F	1	-	-
81	56	M	C	0	-	-
82	78	M	F	1	-	-
83	70	F	N	1	-	-
84	66	F	N	0	-	-
85	65	M	C	0	-	-
86	62	M	F	0	-	-
87	60	F	N	1	-	-
88	57	M	F	0	-	-
89	76	F	C	0	-	-
90	62	M	C	0	-	-
91	70	F	F	1	-	-
92	67	M	F	0	-	-
93	76	F	F	0	-	-
94	81	F	N	1	-	-
95	79	F	N	1	-	-
96	72	M	C	1	-	-
97	69	M	C	1	-	-
98	62	F	C	1	-	-
99	62	M	C	0	-	-
100	61	F	C	0	-	-
101	59	F	F	0	-	-
102	58	M	F	0	-	-
103	83	F	N	1	-	-
104	77	F	C	1	-	-
105	76	F	F	1	-	-
106	58	M	C	1	-	-
107	76	M	N	1	-	-
108	75	M	C	0	-	-
109	74	M	F	0	-	-

110	70	F	F	2	-	-
111	67	M	F	0	-	-
112	63	M	C	1	-	-
113	71	M	C	1	-	-
114	51	M	C	0	-	-
115	61	M	C	1	-	-
116	78	M	C	0	-	-
117	60	F	C	0	-	-
118	74	F	F	0	-	-
119	71	F	N	0	-	-
120	80	M	F	0	-	-
121	62	M	F	1	-	-
122	75	M	C	2	-	-
123	69	F	C	1	-	-
124	70	F	F	0	-	-
125	67	M	C	1	-	-
126	74	F	C	0	-	-
127	55	M	F	0	-	-
128	59	M	F	0	-	-
129	73	M	F	0	-	-
130	64	F	C	0	-	-
131	63	M	C	0	-	-
132	80	F	F	0	-	-
133	72	M	C	1	-	-
134	75	M	F	1	-	-
135	54	M	C	0	-	-
136	68	M	F	0	-	-
137	55	F	C	0	-	-
138	68	F	F	1	-	-
139	70	F	C	1	-	-
140	79	F	F	1	-	-
141	61	F	C	0	-	-
142	74	F	C	1	-	-
143	76	F	C	0	-	-
144	63	M	N	0	-	-
145	60	F	C	0	-	-
146	65	F	C	1	-	-
147	69	F	C	0	-	-
148	70	M	C	1	-	-
149	77	F	C	0	-	-
150	73	F	N	1	-	-
151	61	M	F	0	-	-
152	65	M	F	1	-	-
153	69	M	N	0	-	-
154	67	M	C	1	-	-
155	66	F	F	1	-	-
156	67	M	F	0	-	-
157	67	M	C	1	-	-
158	80	F	C	1	-	-
159	63	F	C	1	-	-
160	69	F	N	0	-	-
161	62	F	N	1	-	-
162	82	M	F	1	-	-
163	75	F	F	1	-	-
164	62	M	F	0	-	-
165	58	M	C	0	-	-
166	60	F	C	0	-	-
167	65	F	F	1	-	-
168	66	M	C	0	-	-
169	66	M	F	0	-	-
170	62	F	C	0	-	-

171	68	M	C	0	-	-
172	61	F	C	0	-	-
173	69	F	F	0	-	-
174	76	F	C	1	-	-
175	70	F	N	0	-	-
176	74	F	F	1	-	-
177	76	F	C	0	-	-
178	70	M	C	0	-	-
179	65	F	F	1	-	-
180	51	F	N	0	-	-
181	62	F	C	0	-	-
182	60	M	C	0	-	-
183	64	F	F	0	-	-
184	77	F	C	0	-	-
185	71	F	F	0	-	-
186	61	F	F	0	-	-
187	57	M	C	0	-	-
188	69	M	N	1	-	-
189	75	M	C	1	-	-
190	74	F	C	0	-	-
191	65	F	C	0	-	-
192	60	F	C	0	-	-
193	68	F	N	0	-	-
194	60	M	N	0	-	-
195	52	M	C	0	-	-
196	73	F	C	1	-	-
197	68	F	F	1	-	-
198	65	M	C	1	-	-
199	71	M	C	0	-	-
200	75	M	F	0	-	-
201	69	M	C	1	-	-
202	69	M	C	0	-	-
203	72	F	F	0	-	-
204	68	F	C	1	-	-
205	61	M	C	0	-	-
206	56	M	C	0	-	-
207	69	F	C	1	-	-
208	66	M	F	0	-	-
209	77	F	C	1	-	-
210	63	M	C	1	-	-
211	60	M	F	0	**+**	**+**
212	67	M	C	0	-	-
213	67	F	C	1	-	-
214	64	F	C	0	-	-
215	76	M	F	0	-	-
216	77	M	F	1	-	-
217	56	F	C	0	-	-
218	74	M	F	1	-	-
219	59	F	F	0	-	-
220	73	M	F	1	-	-
221	68	M	C	0	-	-
222	75	F	C	0	-	-
223	67	M	C	0	-	-
224	69	M	F	1	-	-
225	74	F	F	1	-	-
226	49	F	C	0	-	-
227	64	M	C	0	-	-
228	64	F	N	0	-	-
229	75	M	F	1	-	-
230	69	F	N	0	-	-
231	64	F	C	0	-	-

232	74	F	F	0	-	-
233	60	M	C	0	-	-
234	49	F	C	1	-	-
235	62	M	C	0	-	-
236	59	F	C	0	-	-
237	65	M	F	0	-	-
238	61	M	F	0	-	-
239	82	M	C	1	-	-
240	72	M	F	0	-	-
241	60	M	F	0	-	-
242	84	M	F	1	-	-
243	65	F	C	1	-	+
244	80	M	F	1	-	-
245	69	F	F	0	-	-
246	58	M	F	1	-	-
247	66	M	F	1	-	-
248	59	M	F	1	-	-
249	67	M	F	1	-	-
250	60	M	F	0	-	-
251	76	M	F	0	-	-
252	68	F	C	0	-	-
253	75	F	N	0	-	-
254	64	F	F	1	-	-
255	62	M	F	1	-	-
256	72	M	N	0	-	-
257	77	M	C	0	-	-
258	65	F	F	0	-	-
259	75	F	C	0	-	-
260	67	M	C	1	-	-
261	66	F	C	1	-	-
262	73	F	F	0	-	-
263	55	F	C	1	-	-
264	55	M	F	0	-	-
265	78	F	N	1	-	-
266	67	F	F	1	-	-
267	54	F	C	1	-	-
268	77	M	F	1	-	-
269	74	M	C	0	-	-
270	66	F	C	1	-	-
271	65	F	C	0	-	-
272	67	F	C	0	-	-
273	66	M	C	0	-	-
274	59	M	F	0	-	-
275	63	F	C	1	-	-
276	76	M	F	1	-	-
277	60	M	C	0	-	-
278	66	F	C	0	-	-
279	62	F	C	0	-	-
280	62	M	C	1	-	-
281	68	F	C	1	-	-
282	77	M	C	1	-	-
283	63	M	C	0	-	-
284	64	M	C	1	-	-
285	48	F	F	1	-	-
286	72	M	C	0	-	-
287	64	F	C	1	-	-
288	67	F	C	0	-	-
289	57	F	N	0	-	-
290	73	M	F	0	-	-
291	79	M	F	1	-	-
292	73	F	F	0	-	-

293	76	F	F	0	-	-
294	67	M	F	1	-	-
295	67	F	C	1	-	-
296	73	F	F	0	-	-
297	60	F	F	0	-	-
298	64	F	N	1	-	-
299	65	F	C	0	-	-
300	70	M	C	1	-	-
301	79	F	F	1	-	-
302	45	F	N	0	-	-
303	70	M	F	0	-	-
304	68	F	N	0	-	-
305	76	M	N	0	-	-
306	58	M	F	0	-	-
307	62	F	C	0	-	-
308	65	F	F	0	-	-
309	51	F	C	0	-	-
310	69	F	F	0	-	-
311	57	F	F	0	-	-
312	71	F	C	1	-	-
313	74	M	F	1	-	-
314	62	M	C	1	-	-
315	76	F	F	0	-	-
316	62	F	F	0	-	-
317	61	M	F	0	-	-
318	54	F	N	0	-	-
319	42	F	N	0	-	-
320	66	M	F	1	-	-
321	60	F	C	0	-	-
322	74	F	F	0	-	-
323	77	M	C	1	-	-
324	70	M	F	1	-	-
325	64	M	C	0	-	-
326	67	F	C	0	-	-
327	62	F	C	0	-	-
328	74	M	F	1	-	-
329	69	M	F	1	-	-
330	69	M	F	1	-	-
331	69	F	C	0	-	-
332	74	M	F	0	-	-
333	77	M	C	0	-	-
334	67	F	F	0	-	-
335	67	F	N	0	-	-
336	67	M	N	1	-	-
337	66	F	F	1	-	-
338	75	F	C	0	-	-
339	66	F	C	0	-	-
340	62	F	C	0	-	-
341	61	F	C	0	-	-
342	74	M	F	0	-	-
343	71	F	F	1	-	-
344	62	F	C	0	-	-
345	56	F	C	0	-	-
346	69	F	C	0	-	-
347	72	F	C	0	-	-
348	70	F	C	0	-	-
349	69	M	C	1	-	-
350	75	M	C	1	-	-
351	75	F	C	1	-	-
352	70	M	C	0	-	-
353	71	F	F	0	-	-

354	73	M	C	0	-	-
355	71	M	C	0	-	-
356	62	F	C	0	-	-
357	71	M	F	0	-	-
358	77	M	F	0	-	-
359	64	F	C	0	-	-
360	56	F	C	1	-	-
	Mean (67.1 years)	Female (50.8%), Male (49.2%)	C (50.8%), F (37.5%), N (11.7%)	PS 0 (60.0%), PS 1 (38.6%), PS 2 (1.4%)	Positive cases (0.3%)	Positive cases (0.6%)
